# The narrow gap between norms and cooperative behaviour in a reindeer herding community

**DOI:** 10.1098/rsos.171221

**Published:** 2018-02-14

**Authors:** Matthew Gwynfryn Thomas, Bård-Jørgen Bårdsen, Marius Warg Næss

**Affiliations:** 1 Norwegian Institute for Nature Research (NINA), Arctic Ecology Department, Fram Centre, 9296 Tromsø, Norway; 2 Norwegian Institute for Cultural Heritage (NIKU), High North Department, Fram Centre, 9296 Tromsø, Norway

**Keywords:** evolution of cooperation, kinship, in-group versus out-group behaviour, field experiments

## Abstract

Cooperation evolves on social networks and is shaped, in part, by norms: beliefs and expectations about the behaviour of others or of oneself. Networks of cooperative social partners and associated norms are vital for pastoralists, such as Saami reindeer herders in northern Norway. However, little is known quantitatively about how norms structure pastoralists' social networks or shape cooperation. Saami herders reported their social networks and participated in field experiments, allowing us to gauge the overlap between reported and emergent cooperation. We show that individuals' perceptions of reciprocal cooperation within their social networks exceeded actual reciprocity, although both occurred frequently and were concentrated within herding groups. Herders with more extensive cooperation networks received more rewards in an economic game. Although herders overestimated reciprocal helping, cooperation in this community was still extensive, suggesting that perceived norms potentially allow network structures promoting cooperation to emerge and be maintained.

## 1. Introduction

Social networks are vital for pastoralists, who rely on combinations of kin, herding group members, and socially distant others for cooperation, production and survival [1,2]. Norms help shape social worlds, in turns mandating and constraining behaviour [3]. Some pastoralists follow norms of sharing based on transfers to those in need [4,5] that function to pool risks in an uncertain environment, leading to greater survival and wealth equality [6]. Institutions can also guide norms and behaviour; for example, wealthier Orma pastoralists contributed more to a public goods experiment after recognizing superficial similarities to a local fundraising institution [5]. Despite scattered evidence that social norms and institutions influence the behaviour of individual herders, little is known about how espoused norms structure pastoralists' social networks, affecting actual cooperation.

Norms are socially learned beliefs or expectations about common or uncommon behaviour (descriptive norms), or prescribed and proscribed behaviour (injunctive norms) [3]. People tend to be aware of their group's norms and where they stand in relation to them, and individual preferences need not be consistent with these norms [7]. Wallen & Romulo [8] distinguish between actual behaviours occurring and ‘normative social beliefs’, which are individuals' perceptions of typical or accepted behaviour (either their own or the behaviour of others). Here, we investigate descriptive norms.

There is often a disjuncture between what people do and what people say they do. Compared to self-reported behaviour, people consume more alcohol [9], wash their hands less frequently [10], exercise less [11] and are less likely to vote [12]. In some cases, actual behaviour can positively but imperfectly correlate with norms; for example, regarding normative judgements about impulse purchases [13] or the risky behaviour of peers [14].

Fairness, reciprocity and loyalty to one's in-group(s) have been posited as fundamental aspects of human morality [15]. However, ethnographic evidence from smaller-scale societies reveals gaps between norms of sharing or reciprocity and resultant cooperative behaviour. Inuit people in north-western Canada equated generosity with prestige and held the idea that exchanges should be reciprocal and roughly balanced. A quantitative analysis of hospitality (in terms of gifts and visits) found that low-status individuals were generous and regular gift givers, while the more productive families offered conspicuous, larger but irregular giveaways [16]. Similarly, the ideal that exchange should be balanced meant it was unnecessary for villagers to keep accounts of transfers, allowing imbalances in visits and gifts to be maintained without punishment. The authors observed: ‘What people said about the rules of sharing sometimes counted for more than what people did’ [16], p. 235].

Pastoralist households in Namibia transfer food to others on demand through a norm of sharing [17]. Although people sometimes attempt to conceal the amount, types or quality of food they possess, the heat forces them to cook outside, presenting opportunities for demand sharing and increasing the social costs of excluding people [18]. Despite this sharing norm giving everyone the right to demand and receive food from anyone, reciprocated transfers were higher than expected if sharing were truly widespread and unconditional. Reciprocal relationships emerged within spatial clusters around households, especially for high-value goods, and did not appear to be influenced by kinship [17].

Pastoralists exhibit a greater tendency to learn socially from one another compared with more sedentary and individualistic groups [19], which may encourage the spread of norms within herding communities. Transmission of norms and the cooperative acts emerging from adherence to them (or not) can be modelled as a social network involving links between individuals. Individuals have direct connections with others, representing dyadic interactions. Strong dyadic ties support the evolution of cooperation in a wide range of network structures [20] and the freedom to choose social partners by forming or dissolving ties can also boost levels of cooperation [21,22]. Previous studies of the presence and practice of norms have not considered factors such as social group membership or kinship in social network formation [17] or have only included relationships between groups, not within them [16].

Here, we examine pastoralists' descriptive normative social beliefs (i.e. perceptions of how they and others cooperate) and the extent to which their beliefs correspond to actual cooperative behaviour. We also investigate the relative and mutually inclusive importance of kinship and herding group membership in structuring patterns of cooperation, analysing herders' direct social bonds to quantify the extent to which cooperation is bounded by herding group membership. From these goals, we developed five hypotheses:

H1. based on the literature reviewed above, we expect that normative social beliefs about reciprocity (i.e. self-reported two-way social ties) will be more prevalent than the actual reciprocal ties emerging from the complete network of cooperation;
H2. owing to the reliance on cooperative herding groups [2,23], we predict that herders will preferentially name members of their own herding groups in their reported cooperation networks and give gifts to them in an economic game, leading to high assortment on group membership;
H3. this pattern of gift giving will also lead to a strong similarity between the gift network and the reported cooperation networks;
H4. overall, we expect to find a strong reliance on herding group members and close relatives [24,25]; and
H5. previously, we found that approximately one-third of gifts were given to non-kin belonging to other herding groups [24], suggestive of cooperation based on reciprocity and/or reputation but not kin selection. If reputation based on herding ability is an important driver of cooperation, we predict that people who were reported by others as having more ties in the cooperation network will receive more gifts.

To test these hypotheses, we conducted interviews and field experiments with Saami reindeer herders using a large winter district in Finnmark, northern Norway. Reindeer herders—a minority of the Saami ethnic group—organize their labour into *siidas*: groups consisting of household members comprising kin and non-kin that are associated with particular pastures and can change composition seasonally [23]. Here, we focus on cooperation in winter siidas, where there tends to be lower trust and more conflicts compared with summer siidas [26]. Further details about the study site and data collection are presented in the electronic supplementary material.

## 2. Material and methods

Interviews and economic games were conducted between June and August 2016 by the first author and a field assistant; see the electronic supplementary material, figure S1 for location of the study site. Surveys, game scripts, details of data transformations and other materials are deposited in the Open Science Framework (OSF; https://osf.io/9sn6k/). We performed a confirmatory study where our working hypotheses were pre-registered prior to our data collection.

### 2.1. Mapping cooperative networks

**Figure 1.**
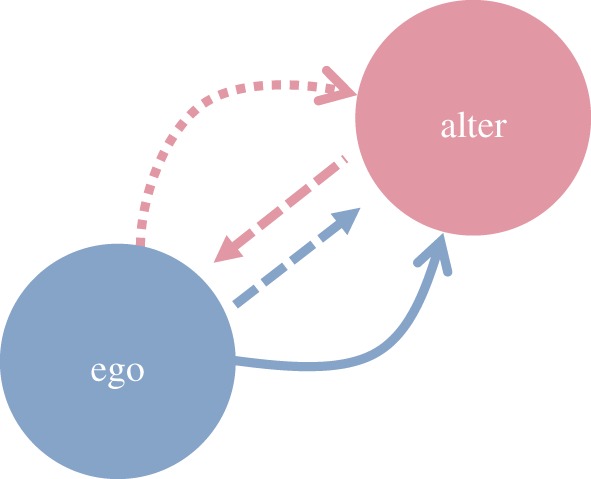
Measures of cooperation used in this study. Cooperation given from ego to alter, as reported by ego (blue solid arrow) and as reported by alter (pink dotted arrow); and gifts given from/to ego and alter (dashed arrows).

We adapted the Net-Map method [27] for participatory mapping of social networks. Participants drew ego-centred social networks for three kinds of cooperation: advice, help and sharing items. Each participant could freely choose whom they wanted to include, the only restriction being the person they named had to hold a licence for reindeer herding (although in practice, several participants also named collections of people; these were dropped from the analyses presented in this paper). Ties were directed, allowing for the possibility that ego reports cooperating with alter but not receiving cooperation in return. For each ego–alter pair, we can quantify whether cooperation was given, as reported by ego and by alter (figure 1).

### 2.2. Kinship ties

Participants indicated their genealogical relatedness to each person in their cooperative network through a sequence of immediate kin (gendered parent, child or sibling) ties. For example, if ego and alter were brothers, this would be indicated by ‘B’; if alter was ego's father's brother's son (first cousin), this was recorded as ‘FBS’. We then calculated coefficients of relatedness [28] based on the number of immediate kin ties (*k*): *r *= 1/2*^k^*. We also asked participants to report parents, siblings and children who were licence owners if they were not already drawn on the social network maps.

### 2.3. Gift games

We endowed participants with 35 l of petrol, priced at 15 NOK per litre, giving a total endowment of 525 NOK ($52.34 purchasing power parity [PPP] in July 2016). Participants were asked to give everything away to at least one other licence owner, up to as many recipients as they desired. For each gift, we recorded the anonymized ID number of the recipient, the amount given and a *post hoc* reason for the gift. We aggregated the set of allocation decisions into a network of gifts for the district. All gifts were given anonymously and payments were lumped into the total amounts earned, which were paid via bank transfer at the end of the data collection period. Thus, no herders knew how many gifts they received or from whom they came.

The question of external validity—whether behaviour during experiments generalizes to real-world situations [29–31]—is important for researchers to consider. We aimed to make our experimental game externally valid by using petrol as the currency, rather than cash, because participants were making allocation decisions based on a commodity that is important in a reindeer herding lifestyle, while still allowing experimental control.

Note that the game structure presented here differs from previous studies employing gift games, which limited the number of potential gift recipients to three [24,32–34]. We endowed participants with a larger sum than usual in this game and did not limit the number of recipients; thus, our formulation of this economic game can capture social networks at a finer scale of resolution compared to previous studies.

### 2.4. Statistical analysis

All analyses were conducted in R 3.3 [35].

#### 2.4.1. Similarity between social networks

We calculated Jaccard indices [36] to quantify the similarities between the advice, help, sharing and gift networks. A Jaccard index *J*(*A*, *B*) (where 0≤J(A,B)≤1) measures the proportion of links shared between binary networks *A* and *B*, compared to the links in either network. Indices closer to 1 indicate greater similarity.

#### 2.4.2. Predicting ties in the cooperation network

We fitted a social relations model (SRM) using an iterative Markov chain Monte Carlo algorithm implemented in the ‘amen’ R package [37] to understand the relationship between normative cooperation (naming others in the cooperation networks: the outcome variable) and cooperative behaviour (gift giving), herding group membership and kinship. SRMs have a long history as a tool for analysing dyadic data and have recently been used in other anthropological studies of cooperation in small-scale societies [38–40].

SRMs partition the variance in dyadic observations into giver, receiver and relationship variance components. We fitted a random effects model with a probit-link (see the electronic supplementary material, table S1 for specifications) predicting whether herder *i* named herder *j* in their cooperation network (*y_i_*_,*j*_):

$$\begin{array}{rl} z_{i,j} & =\beta_{d}^{T}x_{d,i,j}+\beta_{g}^{T}x_{g,i}+g_{i}+r_{j}+d_{i,j}\\ \;\text{and}\;y_{i,j} & =1(z_{i,j}>0), \end{array}$$

where ***x****_d_*_,*i*,*j*_ is a vector of predictors for dyad {*i*, *j*} and ***x****_g_*_,*i*_ is a vector of ego covariates (in this case, a dummy variable representing whether ego was interviewed). Giver, receiver and dyadic random effects are captured by *g_i_*, *r_j_* and *d_i,j_*, respectively; all random effects are mean-zero bivariate normally distributed.

The correlation between giver and receiver effects measures the extent to which ties in cooperation networks are generally reciprocated. The dyadic (co)variance allows us to calculate dyadic reciprocity: the extent to which ties in the cooperation network were reciprocated owing to the unique features of particular relationships, beyond giver and receiver effects. We calculated variance partition components to quantify the relative importance of egos, alters and their relationships as sources of variation.

In the absence of formal model comparison techniques available in the ‘amen’ package, we assessed model fit by comparing (1) four goodness-of-fit statistics across models and (2) two performance metrics. The goodness-of-fit statistics were: (i) standard deviations of ego means; (ii) standard deviation of alter means; (iii) dyadic correlations; and (iv) triadic dependence. For each of these four measures in each model, we calculated the root-mean-square error (RMSE) between the posterior predictive distribution and the observed network statistics. The model with the lowest RMSEs across the four measures was considered the best fitting (electronic supplementary material, table S1). We also compared models' posterior predictions to observed connections in the cooperation network to calculate F_1_ scores (the harmonic mean of a model's precision and recall) and accuracy (the proportion of true results predicted by the model); see the electronic supplementary material, figure S4. Overall, the additive model—relatedness + siida membership + gifts—provided a marginally better fit compared to other specifications. As it also was the simplest model, we selected it and used this model for making inference.

Further technical details and a discussion of the model selection procedures are presented in the electronic supplementary material.

#### 2.4.3. Cooperativeness and gifts received

We fitted Bayesian Poisson regressions to investigate how the number of times a herder was named as a cooperative partner (in-degree) predicted the number of gifts they received. A univariate model was compared to an intercept-only specification and a negative binomial model. Model weights were calculated by stacking their predictive distributions [41]. From the best fitting model, we calculated 95% credible intervals—measuring uncertainty in the linear model—and simulated data from the posterior predictive distribution to generate predicted outcomes for interesting values of the predictors (shown as 95% prediction intervals) [42]. See the electronic supplementary material, methods for further details.

## 3. Results

We interviewed 33 herders, 31 of whom held government-granted licences to keep reindeer. Thirty of the 31 licence owners played a gift game in which they were endowed with 35 l of petrol and asked to give everything away to at least one another licence owner in their district (see Methods; one person did not play the gift game owing to time constraints that meant his interview terminated early). Participants gave 76 gifts to 44 people (see the electronic supplementary material, figure S2 for distribution of gifts). The median amount given was 8.75 l of petrol (approximately NOK 131.25, or US$ 13.09 PPP at the time of study). The median amount received in total was 18.12 l (NOK 271.8; $27.10 PPP); the largest amount received by any one herder was 81.67 l (NOK 1225.05; $122.14 PPP).

Twenty-seven licence owners drew social networks representing their cooperation partners (see Methods for details). Several participants mentioned groups or categories of people (e.g. ‘elders in the winter siida’ or ‘members of neighbouring siidas’) in their networks, which were subsequently dropped from the quantitative analysis of individuals presented here. After removing all entities in the networks that were not licence owners, we were left with 26 networks naming 60 licence owners, 82.4% of whom belonging to the same siida as the interviewee.

**Figure 2.**
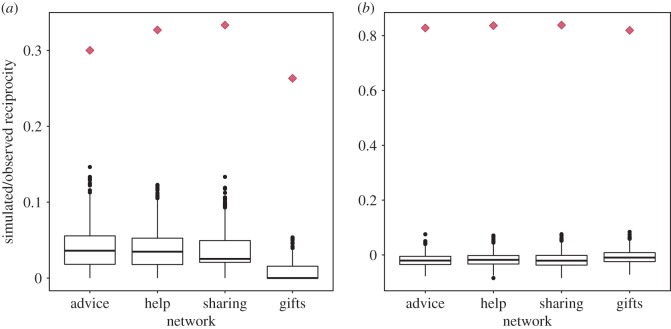
Reciprocity and assortment in herders' social networks: (*a*) reciprocity and (*b*) assortment in the self-reported advice, help and sharing networks, and the gift network. Diamonds show observed network statistics; boxplots are the distributions of reciprocity and assortment in 1000 random networks with the same numbers of nodes and edges as the observed networks.

Participants reported a strong perceived descriptive norm of reciprocity: every person we interviewed said they received advice, help or items from each licence owner they reported helping in turn. Taking the aggregated advice, help and sharing networks, and analysing only people we interviewed as egos and alters, 70.6% of links were reciprocated. This suggests that actual reciprocated cooperation is somewhat lower than the espoused descriptive norm (supporting H1), although reciprocity is still high in this community. Every reciprocated link was with a member of the same siida. Reciprocity was slightly higher in the three self-reported cooperation networks (range: 0.3–0.333) than that in the gift network (0.263; figure 2*a*). There was strong assortment with members of the same siida in all four networks (range: 0.819–0.839; figure 2*b*; supporting H2).

The advice, help and sharing networks each had approximately 45% of their connections in common with the gift network (electronic supplementary material, table S2), suggesting they are structurally similar, as predicted by H3. As the advice, help and sharing networks were very similar to one another, for the remaining analyses we aggregated these three measures into a single binary network of self-reported cooperation (hereafter: cooperation network).

**Figure 3.**
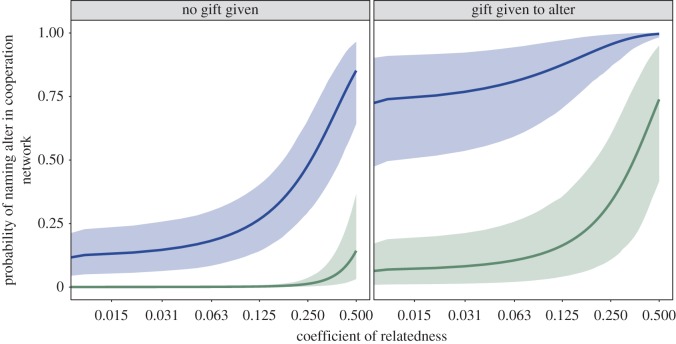
Predicted probabilities of being named in the cooperation network. Probabilities were calculated from the best-fitting social relations model and are conditional on the dyad's relatedness (*x*-axis), whether they belong to the same herding group (blue) or not (green), and whether ego gave a gift to alter (right panel) or not (left panel). Lines represent medians from the posterior predictions and ribbons are 95% credible intervals.

We fitted an SRM to estimate the probability of one herder naming another in their cooperation network, for all pairs of herders (dyads) in the district. The SRM is a useful tool for analysing social networks because it partitions the variance in an outcome into separate components for givers, recipients and relationships. SRMs also calculate reciprocity correlations in giving and receiving (see Methods and references therein for further details). Members of the same herding group were more likely to be named in cooperation networks (odds ratio = 9.384; 95% credible interval [6.506, 15.025]; electronic supplementary material, table S3), regardless of relatedness and whether the participant also gave a gift. Closer kin were more likely to be named as cooperators compared to distant relatives and non-kin (figure 3). Members of the same siida who also received gifts had the highest probability of being named in cooperation networks, regardless of kinship. These results support H4.

The generalized and dyadic reciprocity correlations estimated by the SRM are uncertain (electronic supplementary material, figure S3*a*) but match observed reciprocity in the networks. Positive values for generalized reciprocity suggest that herders who name more people in their cooperation networks tend to be named more often in return; positive dyadic reciprocity suggests that when one herder names another in their cooperation network, the link tends to be reciprocated. Over half of the variance in ties in the cooperation network was explained by dyadic reciprocity (55.03 ± 9.07%), meaning that the unique relationships between egos and alters in dyads provide the main source of variation. The variance component for givers explained 30.18% (±9.48%) and receivers explained 12.42% (±5.43%) of the variance (electronic supplementary material, figure S3*b*), suggesting that 42.6% (i.e. 30.18 + 12.42%) of the variance in naming people in cooperation networks was owing to herders' roles as people doing the naming and people being named.

**Figure 4.**
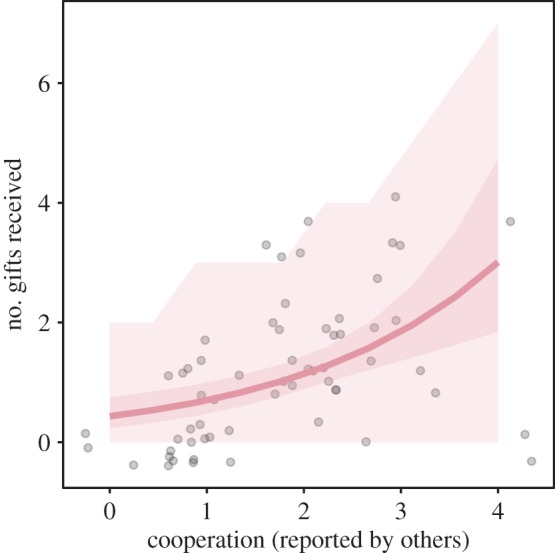
Reputation weakly predicted gifts received. Number of gifts received by herders is predicted by their reputation for being cooperative (measured as the number of times they were reported as helping in other herders' cooperative networks). Pink line shows the median; darker pink shaded area is the 95% credible interval; lighter pink shading is the 95% posterior prediction interval.

Herders reported extensive cooperation networks (electronic supplementary material, figure S4). Herders with more extensive cooperation networks (i.e. those who reported helping, advising and sharing items with more people) received more gifts (figure 4; weakly supporting H5).

## 4. Discussion and conclusion

Our study shows that descriptive normative beliefs about the extent of reciprocal cooperation within a reindeer herding district exceeded actual levels of reciprocity, as evidenced from the aggregation of reported networks and gift giving behaviour in an economic game. Herders reported reciprocal relationships with every social partner, all of whom belonged to the same siida as him or her. High reciprocity also emerged from analysing the proportion of herders who independently named one another in their social networks. Reciprocity was slightly lower in the gift network, although still above the level expected in random networks (despite gift decisions being anonymous and uncoordinated). These patterns suggest that herders overestimated the prevalence of reciprocity among their social partners, although reciprocated cooperation still occurred frequently in the aggregate at a similar level to that observed in other small-scale societies [34,43].

It is possible that participants were exhibiting a social desirability bias to the data collectors by over-reporting the extent of reciprocity among their herding partners so as to appear more collaborative. While we cannot explicitly test for this bias given our data, it is unlikely that every single participant would show the same bias to the same degree and report 100% reciprocity within every social network; normative social beliefs about reciprocity provide a more parsimonious explanation. Regardless, high rates of reciprocity and assortment emerged from the aggregated cooperation networks and the gift game, suggesting that these mechanisms are important for cooperation among reindeer herders, as found across a wide range of human societies [44].

Cooperation was mainly centred on members of the same winter herding groups, especially close kin, with whom the herders engaged in reciprocal and assortative relationships; this pattern matches previous quantitative evidence for the importance of summer siidas [24,25] and supports older ethnographic observations [23]. In addition, as in a previous quantitative study of reindeer herders' cooperation [24], we found an additive rather than interactive effect of relatedness and group membership on cooperation suggesting they are two independent factors driving cooperation. Future work should attempt to explicitly link the cooperative networks studied here to the local ecology, encompassing changes in pasture use and land tenure regimes.

Reciprocity is but one factor affecting the evolution of fairness norms, along with population structure, noise, spite, empathy and reputation [45]. We inferred a herder's reputation from the number of times they were named in the social networks of others. Herders with better reputations (i.e. more ties reported by others) received more gifts. Extensions of this study should attempt to operationalize more concrete measures of reputation, rather than inferring reputation from ties in social networks. As part of the social network mapping methodology adapted for use here (see Methods), we attempted to gain an insight into reputation by asking participants to rate the influence of people they named in their social networks. Despite anonymizing these networks by using identification numbers in place of names, participants were uncomfortable with this task and we dropped it early in the data collection process.

We used a gift game framed in terms of petrol, a commodity used by reindeer herders for day-to-day tasks and large-scale seasonal migrations involving intensive periods of collaboration [24]. Arguably, gift giving in this economic game is not a measure of cooperation, as defined by some evolutionary scientists [46] (but not others [47]): allocation decisions were costless and free of dilemmas. Regardless, the network of gift decisions effectively reveals information about existing social relationships, allowing some insight into the patterns of cooperation in this community. The strong association between gift giving and the reported cooperation networks is unlikely to be owing to framing, in the sense that participants might donate more readily to people they had already named in their networks. Interviews lasted on average 1.5 h (some taking up to 3 h), with social network mapping occurring near the start of the process and gift game decisions at the end. In addition, participants' social network diagrams were packed away well before the gift games commenced.

Failure to abide by norms can lead to punishment in some contexts, or even self-punishment such as guilt, anxiety or embarrassment in cases when norms become more internalized [48]. Theoretically, the internalization of norms—where following a norm becomes a goal in itself—readily evolves, allowing cooperation to become more ‘instinctive’, especially when peer-punishment of free riders occurs [49]. It is important to understand the factors that cause individuals to deviate from their perceived norms. Following norms is not an all-or-nothing behaviour: there might be shades of conformity, whereby people behave nearer or further from the average or ideal behaviours in their population [7]. Spatial constraints and individual differences (e.g. in resource acquisition ability or other characteristics determining a potential social partner's ‘market value’ [50]) might play a role; both can lead to particular subsets of relationships emerging from norms of widespread sharing, which would otherwise predict fully connected, but not necessarily reciprocal, cooperative networks [17].

This study provides the first quantification—as far as we are aware—of the gap between a cooperative norm and its associated behaviour. Our results suggest there is a gap, albeit small, between the normative social beliefs of individual pastoralists and actual occurrences of cooperation, with pastoralists reporting an optimistic social world of perfect reciprocity. Perceived social norms relating to cooperation, and the cooperative acts themselves, centred on members of the same herding group, especially closer kin. Previous studies did not explicitly quantify this gap or explore cooperation in the light of relatedness and group membership. A study of Inuit villages found that social status affected the cooperative behaviours emerging from a norm of widespread, balanced reciprocity [16]. Status does not appear to play a role in Saami groups owing to herders' egalitarianism when it comes to decision-making and cooperation.

People overestimate reciprocity, though not by much. The concordance between perceived and actual cooperation implies that social norms play a role in maintaining social network structures that encourage cooperation as much as they encourage such network structures to form in the first place. An alternative hypothesis is that social constraints (e.g. almost all herders in our study district did not move to new winter groups during the last 15 years) limit the formation and dissolution of ties in a network, potentially requiring hyper-cooperative norms to evolve in order to stop universal defection. This effect might also be driven by competition between groups for pastures. To begin disentangling these hypotheses, researchers should seek to understand the dynamic interplay between the evolution of norms and institution, the subsequent patterns of cooperative interactions, and how each affects and is affected by individuals.

## Supplementary Material

Supplementary Information for ‘The narrow gap between norms and cooperative behaviour in a reindeer herding community‘

## Data accessibility

The anonymized, cleaned and processed data used for analyses presented here are available from Dryad (http://dx.doi.org/10.5061/dryad.g44g3) [51]. R code to reproduce the analyses is available from: https://github.com/matthewgthomas/saami-norms.
