# Supplementary material for: The narrow gap between norms and cooperative behaviour in a reindeer herding community
Source: R Soc Open Sci. 2018 Feb 14;5(2):171221. doi: 10.1098/rsos.171221 (PMC5830731; doi:10.1098/rsos.171221)
Supplement: Supplementary Information for ‘The narrow gap between norms and cooperative behaviour in a reindeer herding community‘ [file rsos171221supp1.docx]

# Supplementary Information for ‘The narrow gap between norms and cooperative behaviour in a reindeer herding community’

Matthew Gwynfryn Thomas, Bård-Jørgen Bårdsen, Marius Warg Næss

## Overview of reindeer husbandry and the study site

Reindeer pastoralism likely began in the Arctic regions of Norway, Sweden, Finland and westernmost Russia at least 400 years ago^1^. Since the 1960s, the methods of production have been increasingly assisted by modern technologies (including snowmobiles, all-terrain vehicles, helicopters, GPS, mobile phones, drones and social media [e.g. using Facebook to share information and coordinate activities]) and the lifestyle has shifted to being a more market-oriented industry, with concomitant sedentarisation for the herders. Today, Saami people—an ethnic group indigenous to the area—have the right to own and herd reindeer in Norway, and are afforded protection under the International Labour Organization’s Indigenous and Tribal Peoples Convention, 1989 (No. 169)^[[1]](#footnote-1)^.

Saami reindeer herders’ social organisation is comprised of three layers. The basic unit is the ‘siida share’ (Norwegian: *siidaandel*): a license granted by the government entitling the owner to manage a herd of reindeer within a designated area. (Henceforth, we will refer to siida shares as license owners.) One or more license owners belong to at least one *siida*, which is a cooperative herding group composed of households and traditionally organised around kinship (although today, siidas include non-kin). Siidas are grouped into districts: formal administration units defined by the government^2^.

Our study focuses on Finnmark, the northernmost and largest reindeer herding area in Norway, in terms of land use, reindeer populations and number of herders. As of 2016, there were 2,398 reindeer herders and 376 license owners in Finnmark^3^. In Finmark, reindeer migrate seasonally, spending the summer months around the coastal areas or on outlying islands, then moving inland to spend winters on smaller pastures with higher population densities; calving areas are either located on the coast or lie in-between the summer and winter pastures^4^.

These migrations to and from particular areas form the basis of the siida system: the nexus of collective action^1,2,5,6^. Reindeer herders can belong to different siidas depending on the season. Every herder belongs to a summer siida (which is also a legal institution in Norway). These summer siidas typically split into smaller winter siidas based in the interior of the county. Some people also belong to spring/autumn siidas. Winter siidas are the focus of this study.

Pasture access in Finnmark was historically organised around customary access; however, the 1978 Law on Reindeer Herding introduced a ‘common’ (Norwegian: *felles*) land tenure system that arguably was interpreted by some to mean ‘open access’ to pastures and led to competition for land use^7^. This competition might encourage herders to accumulate more livestock in order to secure grazing areas, at the same time potentially excluding others^8^; herd accumulation can also be a rational risk-management strategy for pastoralists in such situations^9–11^.

In an attempt to ameliorate this situation and diffuse what is commonly debated as a tragedy of the commons^7^, the government has mandated policies whose objectives are to achieve ecological, economic and cultural sustainability. In practice, ecological sustainability involves reducing the number of reindeer via subsidies^12^, (enforced) slaughter^9^ and land redistribution^9^. Part of the land redistribution process involves introducing exclusive pasture user rights for siidas (i.e. privatisation) and the concomitant raising of borders between units of land.

At the time of fieldwork, pasture borders in the focal winter district had been decided upon but fences had not yet been raised; despite this, many people acted as if the borders were already enforced. The winter district in which we conducted fieldwork (Figure S1) was used by herders from several summer districts; there were 745 people (95 of whom were license owners) split into 24 winter siidas^3^. We recruited participants by systematically phoning all license owners, the majority of whom were either unreachable, unavailable or unwilling to take part. All participants provided written informed consent.


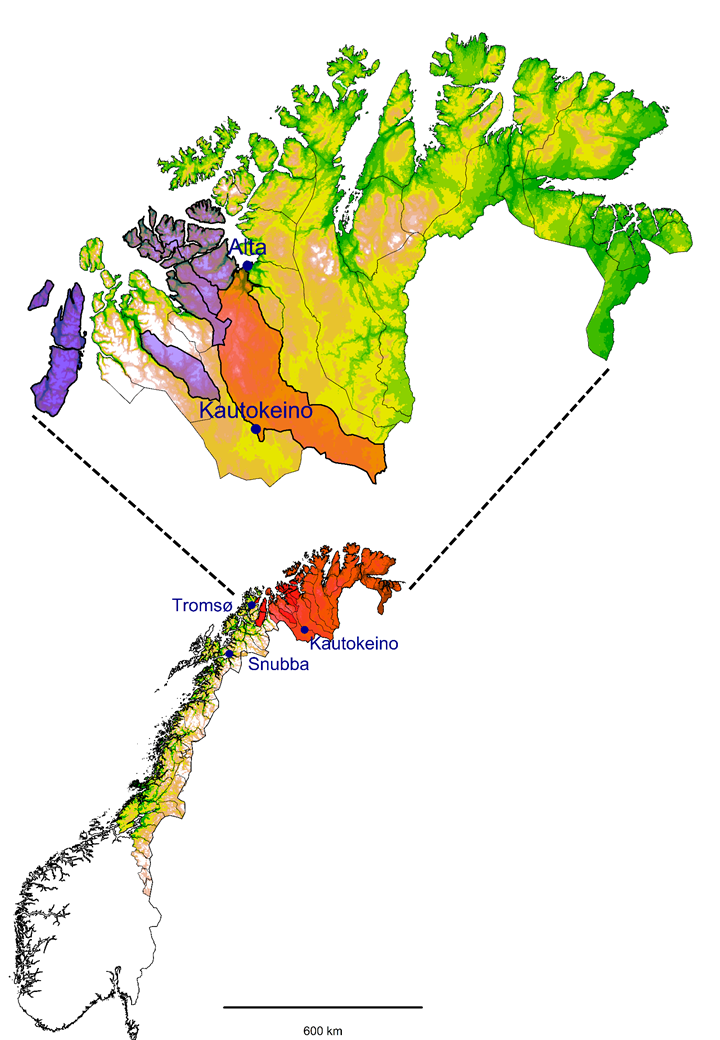


Figure S1: Location of the winter district in which we conducted fieldwork (orange area in inset map), within Finnmark, Norway. Digital elevation mode and country borders: Norwegian Mapping Authorities. Reindeer districts: freely available data from the open internet mapping services ‘Kilden’, a service provided by the Norwegian Institute of Bioeconomy Research: http://www.skogoglandskap.no/temaer/Nedlasting_av_kart (assessed 2017-09-01).

## Supplementary methods

### Social relations model predicting cooperative links

The Methods section in the main text gives the specification for the social relations models we fitted. Following Koster & Leckie^13^, we specified flat (uninformative) priors for all parameters in the social relations model. The individual-level giver and receiver random effects are mean-zero bivariate normally distributed:

$$\binom{g_{i}}{r_{j}}\sim N\left\{ \binom{0}{0},\left( \begin{matrix} \sigma_{g}^{2} & \\ \sigma_{gr} & \sigma_{r}^{2} \end{matrix} \right) \right\}$$

Where $\sigma_{g}^{2}$ is giver variance, $\sigma_{r}^{2}$ is receiver variance, and $\sigma_{gr}$ is covariance between givers and receivers.

The correlation between giver and receiver effects measures the extent that ties in cooperation networks are generally reciprocated: $\rho_{gr}={\sigma_{gr}}/{\sqrt{\sigma_{g}^{2}\sigma_{r}^{2}}}$.

Dyadic random effects are also mean-zero bivariate normally distributed:

$$\binom{d_{i,j}}{d_{j,i}}\sim N\left\{ \binom{0}{0},\left( \begin{matrix} \sigma_{d}^{2} & \\ \sigma_{dd} & \sigma_{d}^{2} \end{matrix} \right) \right\}$$

Where $\sigma_{d}^{2}$ is dyadic variance and $\sigma_{dd}$ is dyadic covariance. Dyadic reciprocity—the extent to which ties in the cooperation network were reciprocated in any particular relationship—was calculated as: $\rho_{dd}={\sigma_{dd}}/{\sigma_{d}^{2}}.$

To quantify the relative importance of egos, alters and their relationships as sources of variation, variance partition components can be calculated as follows:

$$\text{Giver VPC}={\sigma_{g}^{2}}/\left( \sigma_{g}^{2}+\sigma_{r}^{2}+\sigma_{d}^{2} \right)$$

$$\text{Receiver VPC}={\sigma_{r}^{2}}/\left( \sigma_{g}^{2}+\sigma_{r}^{2}+\sigma_{d}^{2} \right)$$

$$\text{Dyadic VPC}={\sigma_{d}^{2}}/\left( \sigma_{g}^{2}+\sigma_{r}^{2}+\sigma_{d}^{2} \right)$$

For model comparison and selection, we used four goodness-of-fit metrics calculated in R’s ‘amen’ package (which also estimates the SRMs’ parameters) and measured the concordance between models’ predictions and observed data by calculating F_1_ scores and accuracies.

The goodness-of-fit metrics calculate four network statistics: standard deviations of (1) the row means and (2) the column means; (3) within-dyad correlations (dyadic dependence); and (4) a normalised measure of triadic dependence (correlations between friends of friends). See the amen package’s documentation for further details^14^. For each, we calculated the root mean square error between the observed statistics and statistics simulated from the models’ posterior predictive distributions.

F_1_ scores are the harmonic mean between a model’s precision and recall, which measure rates of true/false positives/negatives (abbreviated here as TP, FP, TN and FN). Precision, also called positive predictive value, is calculated as:

$$P=\frac{TP}{TP+FP}$$

Recall, also called sensitivity, is:

$$R=\frac{TP}{TP+FN}$$

The F_1_ score ($0\leq F_{1}\leq1$) is:

$$F_{1}=\frac{2\cdot P\cdot R}{P+R}$$

Note that F_1_ scores do not take true negatives into account. This is not a problem for our purposes, since we are concerned with correctly predicting positive cases (i.e. ties in the cooperation network).

Accuracy is the proportion of correct predictions to all cases:

$$Accuracy=\frac{TP+TN}{TP+FP+TN+FN}$$

Note that assessing models’ predictions on the same data used to estimate their parameters runs the risk of selecting an overfitted, complex model. Despite this, the model selection procedures we used all identified the additive model—the simplest specification—as being the best-fitting.

### Predicting gifts

The Bayesian Poisson models were fit using the rstanarm package^15^, running one chain for 10,000 iterations with a discarded warm-up of 2,000 iterations. A univariate model was compared to intercept-only and negative binomial specifications using approximate leave-one-out cross-validation, as implemented in R’s ‘loo’ package^16^.

The model predicting gifts was specified as:

$$y_{i}\sim\text{Poisson}\left( \lambda_{i} \right)$$

$$\log\left( \lambda_{i} \right)=\alpha+\beta x_{i}$$

$$\alpha\sim\text{Normal}\left( 0, 20 \right)$$

$$\beta\sim\text{Normal}\left( 0, 5 \right)$$

Where $\boldsymbol{y}_{\boldsymbol{i}}$ is the number of gifts received by person ***i*** and $\boldsymbol{x}_{\boldsymbol{i}}$ is the number of times they were named in other people’s cooperation networks.

## Supplementary figures


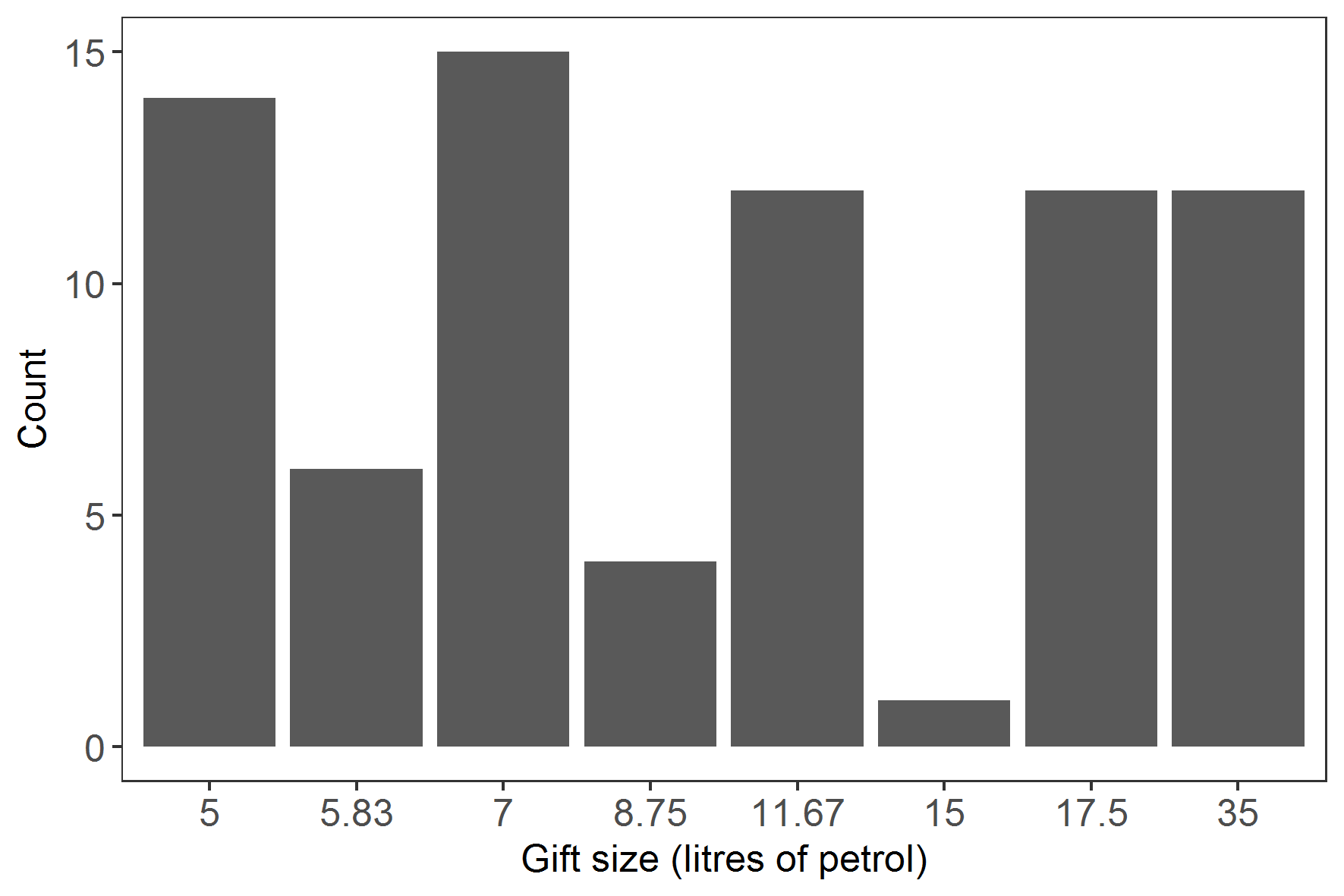


Figure S2: Distribution of gifts in the economic game, in terms of amounts given by each participant.


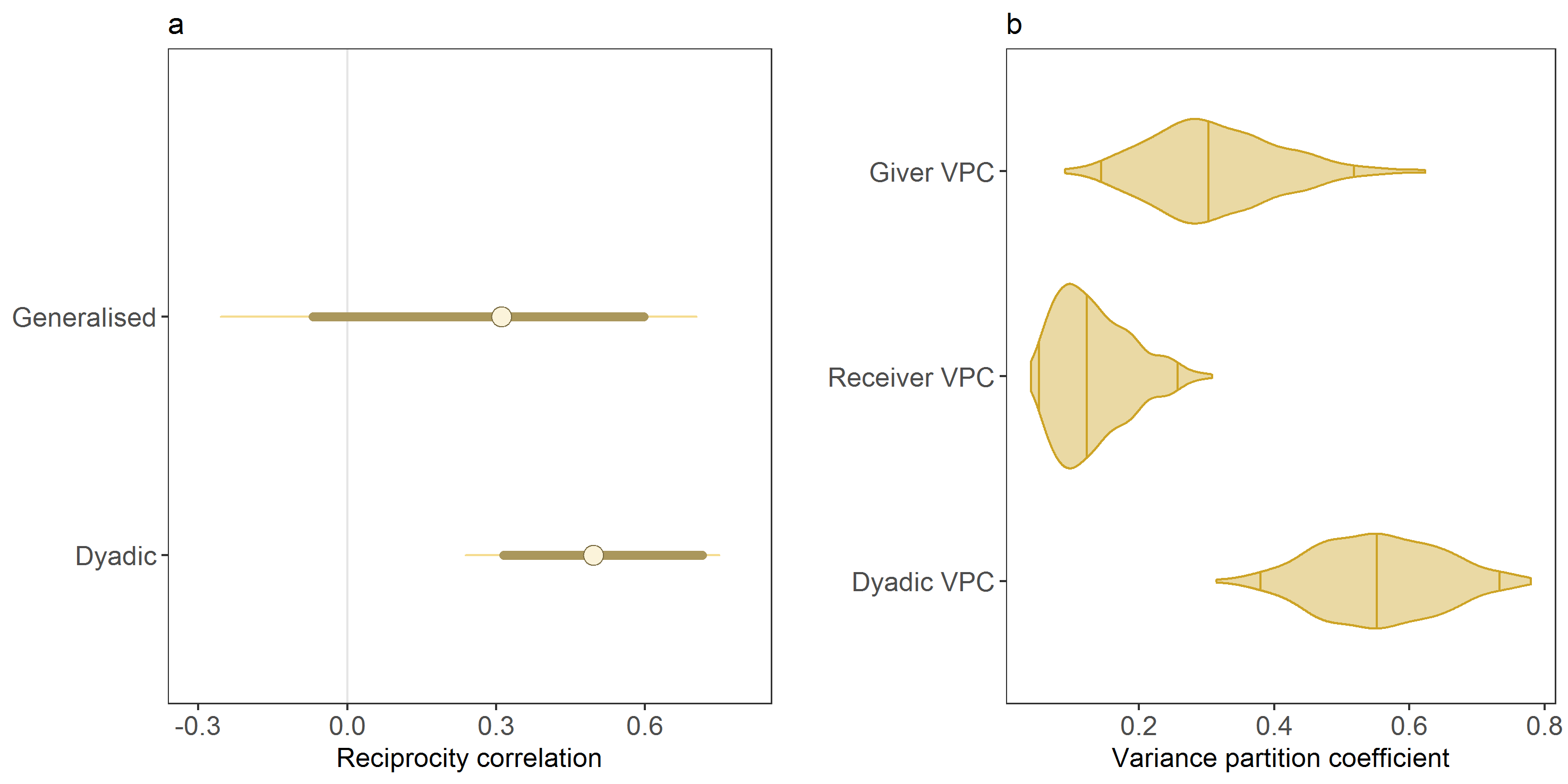


Figure S3: Reciprocity correlations (**a**) and variance partition coefficients (**b**) from the best-fitting social relations model. See Methods for how these values were calculated. Dots in (**a**) show median values from the posterior distributions; thick lines are 80% credible intervals, and thin lines are 95% credible intervals. Lines within the violins in (**b**) represent medians and 95% credible intervals.


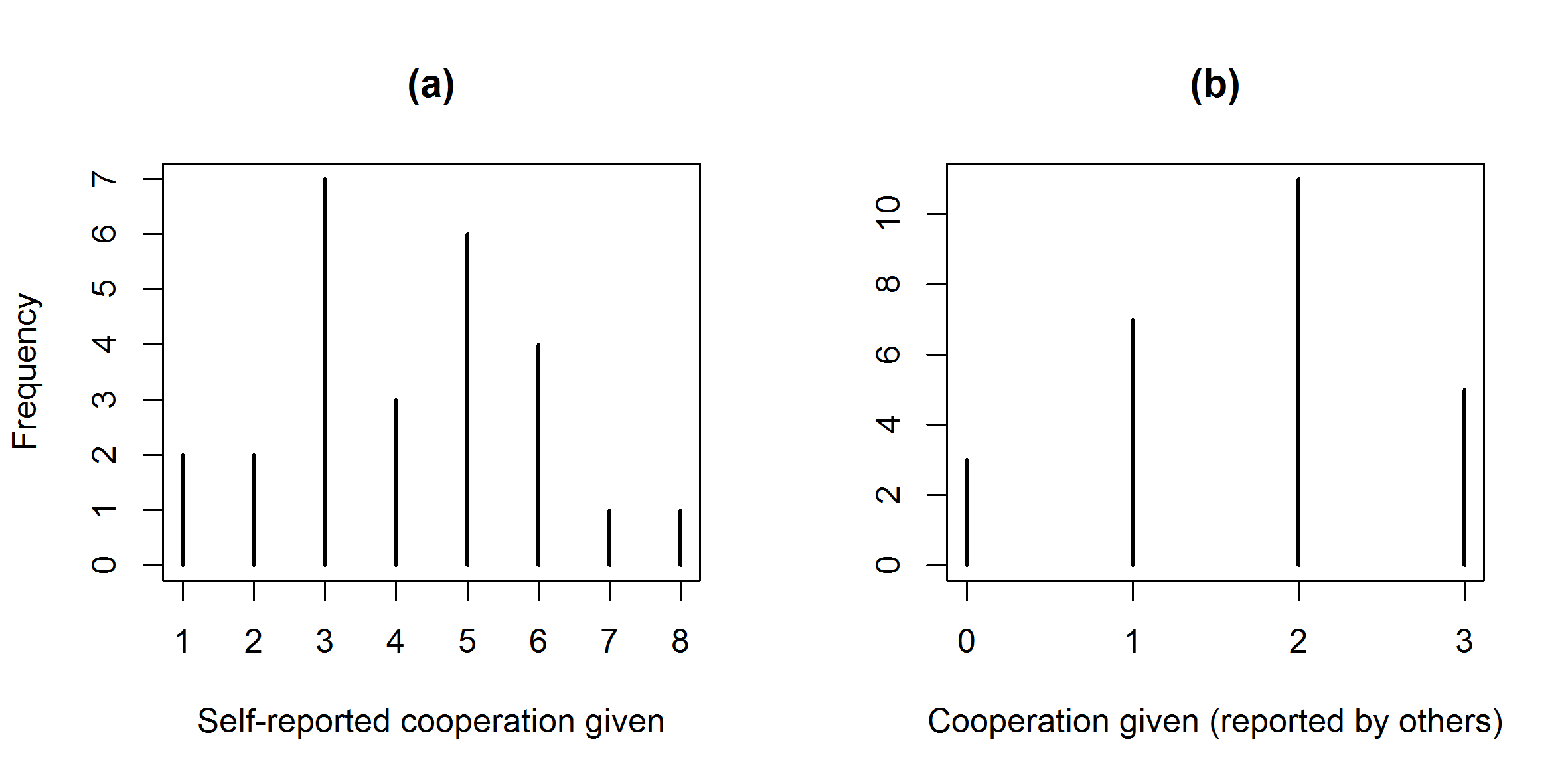


Figure S4: Distributions of number of ties (out-degree) in the cooperation network, as reported by (a) participants themselves and (b) other participants.


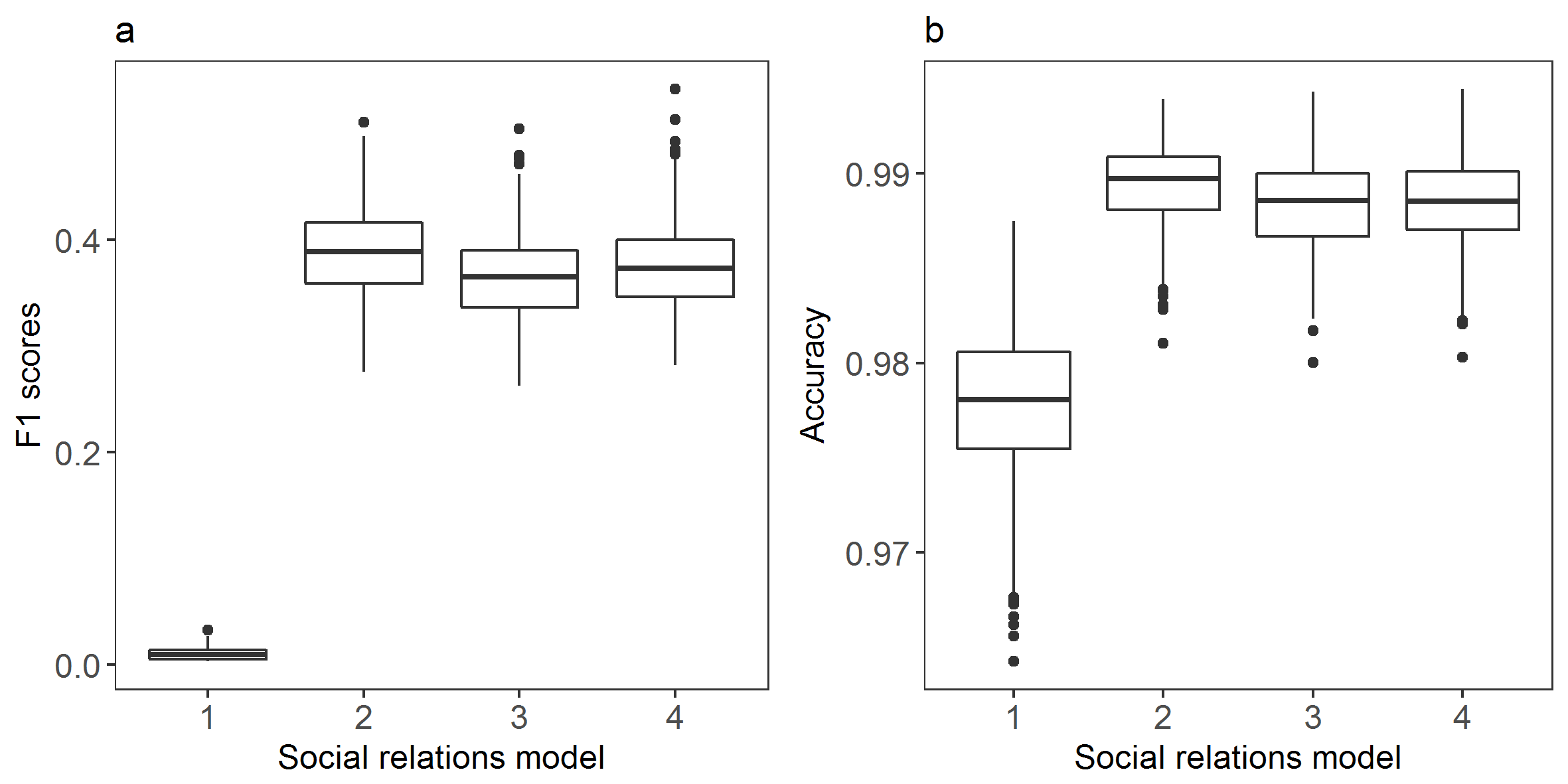


Figure S5: Performance metrics for the social relations models. **a**, F1 score: the harmonic mean of precision (also known as positive predictive value; the ratio of true positives to all positives) and recall (also known as sensitivity). **b**, accuracy: the proportion of true results in the total number cases. Models are (1) intercept-only; (2) relatedness + siida membership + gifts; (3) relatedness × siida membership + gifts; (4) relatedness × siida membership × gifts. Note that all interaction terms include separate additive effects, i.e. ‘relatedness × siida’ means ‘relatedness + siida + relatedness × siida’. Model (2) had the highest median F1 accuracy scores, as well as the best overall goodness-of-fit (Table S1), and is reported in the main text.

## Supplementary tables

Table S1: Root-mean-square errors (RMSE) for each model’s posterior predictive goodness-of-fit statistics compared to observed statistics. Best-fitting models (i.e. lowest RMSEs) for each statistic are highlighted in bold (note that for space reasons, we have only presented RMSEs to four significant digits, which conceals some of miniscule differences between errors). The relatedness + siida membership + gifts model (2) has the best fit as well as best performance (Fig. S4); its results are reported in the main text. Note that all interaction terms include separate additive effects, i.e. ‘relatedness × siida’ means ‘relatedness + siida + relatedness × siida’.

| **Model specification** | **SD ego means** | **SD alter means** | **Dyadic correlations** | **Triadic dependence** |
| --- | --- | --- | --- | --- |
| 1. Intercept-only | 0.0021 | 0.0012 | 0.1011 | 0.0266 |
| 2. Relatedness + siida membership + gifts | 0.0012 | 0.0006 | **0.0562** | **0.0094** |
| 3. Relatedness × siida membership + gifts | 0.0012 | **0.0006** | 0.0707 | 0.0097 |
| 4. Relatedness × siida membership × gifts | **0.0011** | 0.0007 | 0.0621 | 0.0106 |

Table S2: Similarities between the four networks, measured as Jaccard coefficients ($0\leq x\leq1$). Coefficients closer to 1 indicate greater similarity. The correlations suggest that the advice, help and sharing networks are structurally very similar compared to the gift network.

|  | Help | Sharing | Gifts |
| --- | --- | --- | --- |
| Advice | 0.855 | 0.679 | 0.443 |
| Help |  | 0.75 | 0.452 |
| Sharing |  |  | 0.495 |

Table S3: Parameter estimates from the best-fitting social relations model (relatedness + siida membership + gifts). ‘Ego interviewed’, ‘same siida’ and ‘gave gift’ are all binary predictors; relatedness is continuous. All parameters are statistically different from zero.

| **Parameter** | **Log-odds** | **95% credible interval** | |
| --- | --- | --- | --- |
| Intercept | -6.054 | -7.073 | -5.168 |
| Ego interviewed? (ref: no) | 2.587 | 1.873 | 3.373 |
| Relatedness (*r*) | 4.639 | 3.290 | 6.100 |
| Same siida? (ref: no) | 2.239 | 1.873 | 2.710 |
| Gave gift? (ref: no) | 1.847 | 1.139 | 2.549 |
| *r* × same siida | -6.054 | -7.073 | -5.168 |

## Supplementary references

1. Paine, R. *Herds of the Tundra: A Portrait of Saami Reindeer Pastoralism*. (Smithsonian Institution Press, 1994).

2. Thomas, M. G., Næss, M. W., Bårdsen, B.-J. & Mace, R. Smaller Saami herding groups cooperate more in a public goods experiment. *Hum. Ecol.* **44,** 633–642 (2016).

3. Anonymous. *Ressursregnskap for Reindriftsnæringen (Ecological statistics of reindeer husbandry)*. (2016).

4. Bårdsen, B.-J. & Tveraa, T. Density-dependence vs. density-independence - linking reproductive allocation to population abundance and vegetation greenness. *J. Anim. Ecol.* **81,** 364–76 (2012).

5. Thomas, M. G., Næss, M. W., Bårdsen, B.-J. & Mace, R. Saami reindeer herders cooperate with social group members and genetic kin. *Behav. Ecol.* **26,** 1495–1501 (2015).

6. Sara, M. N. in *Reindeer as a Keystone Species in the North* (eds. Soppela, P., Walther, R., Åhman, B. & Riseth, J. Å.) 23–27 (University of Lapland, Arctic Centre, 2002).

7. Marin, A. & Bjørklund, I. A tragedy of errors? Institutional dynamics and land tenure in Finnmark, Norway. *Int. J. Commons* **9,** 19–40 (2015).

8. Næss, M. W., Bårdsen, B.-J., Fauchald, P. & Tveraa, T. Cooperative pastoral production - The importance of kinship. *Evol. Hum. Behav.* **31,** 246–258 (2010).

9. Næss, M. W. & Bårdsen, B.-J. Market economy vs. risk management: How do nomadic pastoralists respond to increasing meat prices? *Hum. Ecol.* **43,** 425–438 (2015).

10. Næss, M. W. & Bårdsen, B.-J. Why herd size matters - Mitigating the effects of livestock crashes. *PLoS One* **8,** e70161 (2013).

11. Næss, M. W. & Bårdsen, B.-J. Environmental stochasticity and long-term livestock viability - Herd-accumulation as a risk reducing strategy. *Hum. Ecol.* **38,** 3–17 (2010).

12. Hausner, V. H., Fauchald, P. & Tveraa, T. The ghost of development past: The impact of economic security policies on Saami pastoral ecosystems. *Ecol. Soc.* **16,** 1–24 (2011).

13. Koster, J. M. & Leckie, G. Food sharing networks in lowland Nicaragua: An application of the social relations model to count data. *Soc. Networks* **38,** 100–110 (2014).

14. Hoff, P., Fosdick, B., Volfovsky, A. & He, Y. amen: Additive and Multiplicative Effects Models for Networks and Relational Data. (2015).

15. Stan Development Team. rstanarm: {Bayesian} applied regression modeling via {Stan}. (2016).

16. Vehtari, A., Gelman, A. & Gabry, J. Practical Bayesian model evaluation using leave-one-out cross-validation and WAIC. *Stat. Comput.* 1–20 (2016). doi:10.1007/s11222-016-9696-4

1. Norway is currently the only country that is home to reindeer herders to have ratified this convention. [↑](#footnote-ref-1)
